# Validation of a Novel Data-Driven Algorithm to Detect Atypical Prescriptions in Radiation Therapy

**DOI:** 10.1016/j.adro.2025.101804

**Published:** 2025-05-10

**Authors:** Connor Thropp, Jaroslaw Hepel, Timothy Leech, Eric E. Klein, Qiongge Li

**Affiliations:** a Department of Medical Physics, Brown University, Providence, Rhode Island; b Department of Radiation Oncology at Rhode Island Hospital, Providence, Rhode Island

## Abstract

**Purpose:**

Erroneous radiation therapy (RT) prescriptions (Rx) can lead to injury or death of patients. A novel data-driven model that uses similarity learning to identify atypical Rx was recently published. In that study, prototype analysis was conducted within a single institution with a single treatment site. The present study sets out to validate the robustness of the model by applying the model to multiple disease sites using a different institution’s data.

**Methods and Materials:**

A query was conducted of Brown University Health RT treatment records for thoracic and brain cancer patients from 1995 to 2021 to create historical databases used for training. The query included records containing data on the Rx and patient-specific features. Simulated anomalies were created to mimic potential errors and were used in the training and testing of the model. Model performance was evaluated using F1 score.

**Results:**

F1 scores for the brain site are 99% for intensity modulated RT, 90% for stereotactic radiation therapy/ radiosurgery/SRT, and 94% for 3-dimensional RT. F1 scores for the thoracic site are 95%, 90%, and 95% for the 3 techniques, respectively. Statistical analysis shows no significant differences between the model’s prediction and ground truth.

**Conclusions:**

The model performance shows feasibility for application to various disease sites across different institutions. This model can be used alongside physicians and physicists during peer review chart rounds to aid in the detection of potential RT Rx errors.

## Introduction

Errors stemming from inaccuracies in the prescription (Rx) for radiation therapy (RT) have a severe impact on clinical outcomes. Overdosing of radiation can lead to grave injuries or fatalities, whereas underdosing can result in suboptimal tumor control.^1^ Many Rx errors go undetected; however, a study by Portaluri et al^2^ exposed 37 RT errors within 5635 treatments, of which 12 were Rx-related. Another investigation by Yang et al^3^ brought to light 7 Rx-related issues during 1262 treatment courses. Physician-led peer-review (PR) chart rounds are the major step of quality assurance to prevent Rx errors from reaching the patient.

PR conducted by physicians represents a quality assurance mechanism aimed at mitigating human error and enhancing patient safety and treatment quality.^4^ However, PR often demands significant time, attention, and physician dedication, frequently yielding inefficient and ineffective results.^5^ Most RT centers hold weekly PR sessions, although some struggle to sustain this frequency because of workload and time constraints.^6^ A significant inefficiency of PR is the time investment required. Studies indicate that the average time dedicated to reviewing an individual patient's treatment plan during PR amounts to approximately 2.7 minutes, escalating to 7 minutes if cases warrant discussion.^5^, ^6^, ^7^ Assuming 40 patients are presented during each PR session, the time allocated for reviewing treatment plans could be as long as 108 minutes per session, with variations based on the extent of peer discussions. Attendance rates in PR mirror the time commitment involved; sessions with fixed timings show an 81% attendance rate, in contrast to 31% for groups without allocated time.^8^ Beyond its inefficiencies, PR also suffers from ineffectiveness. Notably, “hierarchical bias” occurs when senior clinicians' decisions remain largely unchallenged because of their seniority.^8^ Talcott et al^9^ examined PR effectiveness by introducing 20 anomalous cases over 9 weeks, revealing that PR identified only 67% of Rx anomalies. Another study^10^ linked virtual attendance to a 17.7% decline in overall case discussions, including a 10.2% drop in Rx-related discussions.

Anomaly detection is a challenging topic in machine learning because of a few reasons. Firstly, the lack of training samples from the anomaly class creates a significant hurdle for data-driven approaches, because the model needs exposure to anomaly samples to effectively learn.^11^, ^12^, ^13^, ^14^ Secondly, traditional machine learning tools often function as black boxes,^15^, ^16^, ^17^ lacking the capability to provide explanations for flagged anomalies. Thirdly, the creation of simulated anomaly samples often fails to accurately represent real-world anomalies,^18^, ^19^, ^20^ thereby preventing the model from generalizing and becoming less useful in predicting future realistic anomaly events. Li et al^21^ developed an anomaly detection algorithm aimed at overcoming these challenges by developing a rule-based model with clear flagging logic and no requirement for large anomaly data sets during training. Furthermore, in this study, the simulation of anomaly samples was based on conditional probability derived from extensive historical records and real-world anomalies, ensuring that the simulated anomalies closely resemble realistic clinical situations. Combining all the advantages discussed above, this tool could function as a powerful “electronic peer” to assist clinicians during PR.

Although initial testing of the model showed very promising results, it was conducted for a single disease site using only a single institution’s historical database.^21^ In this current study, we set out to apply the model to a new institution’s database as well as new disease sites to test the model’s overall robustness.

## Methods and Materials

### Data acquisition, preprocessing, and feature engineering

Data for this study were obtained through Brown University Health RT medical record system, MOSAIQ (Elekta Corp), with an IRB approval for a retrospective chart review. The query contained approximately 55,300 records of RT treatments from April 1995 to October 2021 that contained patient identifiers, Rx details, and patient-specific features. Moreover, the data collected included diagnostic code, treatment intent, treatment technique, beam energy, age, disease site, dose per fraction, number of fractions, and Rx isodose line, which we used as our feature set. Further preprocessing, discussed in the forthcoming text, reduced the number of historical records to around 2000 and 4100 for the thoracic and brain sites, respectively.

The preprocessing and data extraction phase involved in this work closely mimicked that of our original work.^21^ We aimed to remove records related to quality assurance testing and sought to categorize treatment features, such as modality, technique, and intent, into generalized categories. International Classification of Diseases (ICD-10) were used as a way of extracting site-specific records, as explained in our original work.^21^

Feature engineering techniques were used to impute missing values and reduce the diversity of categorical data. One approach adopted was the “most often” technique, where missing data for the treatment intent and Rx isodose line features were replaced with the most used value for each feature within each Rx for a given model. Additional feature engineering techniques were applied to the patient age data to convert the patient’s age to a categorical age group of either “pediatric” or “adult,” which was used to accentuate the differences between pediatric and adult treatments because of differing radiobiological effects.^22^, ^23^, ^24^ Another data engineering aspect entailed substituting categorical data with weighted numerical values for diagnostic codes. This converted the ICD-10 code into a weighted numerical value, which prioritized frequently used Rx and diagnostic code combinations. This was done by determining the counts of uses for each diagnostic code (ICD-10) within each Rx for a given model. The number of counts was divided by the number of times the Rx occurred in each model, giving a weighted numerical value. This prioritized records with similar Rx and diagnostic codes, and reconditioned the way the model used the feature, as it changed the feature from categorical values to numerical values.

Post engineering processing occurred to minimize excessive flagging of rarely used Rx. For example, reduced field records were intentionally increased in the historical database, increasing their weight. Other rarely used Rx were analyzed by our collaborating physician to determine if the records should be eliminated.

### Model structure

The model used in this work is a distance model that uses 2 dissimilarity metrics to determine the distance between a new Rx and a previously treated Rx, as explicitly described in detail in our original work.^21^ The dissimilarity metrics are the prescription distance (R) and feature set distance (F). R uses Euclidean distance to measure how dissimilar the new Rx is from historically treated Rx, whereas F uses Gower distance^25^^,^^26^ to quantify the dissimilarity of the features associated with the Rx from features of previously treated similar (or same) Rx. The model not only can predict if an Rx is anomalous, but it can also specify why the new Rx is flagged. If R exceeds the threshold for Rx distance, then the record is flagged as a Rx anomaly. Similarly, if F is greater than the threshold for feature distance, then the record is flagged as a feature anomaly. For clarity, an Rx anomaly refers to deviations in the prescribed dose per fraction and the number of fractions, whereas a feature anomaly indicates a deviation in the feature set from those typically associated with that Rx.

### Training and testing

Training and testing required the generation of simulated anomalies. These anomalies were either Rx or feature related and the creation process is described in our original work.^21^ Model training involved the cleaned historical data set relevant to the specific model (brain or thoracic). Sixty records were removed (20 for each technique) for training purposes and would later serve as normal records during testing. For training, each technique (intensity modulated radiation therapy [IMRT], 3-dimensional conformal radiation therapy [3DRT], stereotactic body radiation therapy/stereotactic crainial radiotherapy [SBRT/SRT]) within each treatment site model was provided with 30 simulated anomalies, resulting in a total of 90 anomalies used for each site model. Model training, as discussed in our original work,^21^ determined optimized model parameters which gave the highest-performing F1 scores. Model testing encompassed 20 simulated Rx anomalies, 20 simulated feature anomalies, and 20 normal records for each technique within each treatment site model. The testing parameters were derived from the aforementioned optimization and were specific to each treatment site and technique.

### Validation of results

To validate the model’s predictions, we performed 2 types of validation. First, we gathered a list of 20 correctly predicted anomalies and 10 incorrectly flagged normal records by the model for each treatment site. We blindly provided our collaborating radiation oncologist (RO) with these records and asked him to determine if the records were normal or anomalous. This served to validate our model’s predictions versus those of a physician and to validate that our simulated anomalies were generated appropriately.

Our second form of validation was a statistical analysis using the χ^2^ test. Moreover, we compared the model’s predictions to the ground truth to determine if the model’s predictions were significantly different from the ground truth. We performed the χ^2^ test on the model’s output versus ground truth for all 360 records tested. A contingency table was generated containing a count of the number of predicted anomalies and normal records by the model and counts of the number of anomalies and normal records for the ground truth, which is displayed in Table 1. From the values in the contingency table, we conducted the χ^2^ test to determine if the model predictions were significantly different than the ground truth.

**Table 1:** χ^2^ test results

Metric Type	Anomaly	Normal	Total
Ground truth	240	120	360
Model prediction	264	96	360
Total	504	216	720

We conducted a χ^2^ test to determine the statistical significance of our results. We constructed a contingency table with the total number of anomalous records and normal records for the ground truth and the total number of models predicted anomalies and normal records. We performed the χ^2^ test with an alpha value of 0.05 and found a critical value of 3.84 with 1 degree of freedom. The χ^2^ statistic was determined to be 3.81, meaning we accept the null hypothesis that the model’s predictions are not significantly different from the ground truth.

### Incremental learning

Periodic retraining with new data is necessary to make sure that the model is up to date with current treatment approaches. We tested a linear scaling approach on the thoracic IMRT data set, where records were scaled up in 2-year increments. For example, thoracic IMRT was first used in our clinic in 2006; hence, we applied a scale of 1x to records of those treated in 2006 and 2007; 2008 and 2009 were scaled by 2x, doubling the number of records of those treated during the 2-year interval. This process was repeated through 2021.

## Results

The study's results, including training data and testing data performance using F1 scores and their optimization parameters (a, b, μ, ν, τ, and θ) are summarized in Table 2, along with information on the total historical records (S), simulated anomalies (S_a_), and normal records (S_n_) for training and testing. The fourth column from the right reports the average F1 score and its deviation over 50 runs for testing the training data. Training data testing included 40 normal records and 60 simulated anomalies per treatment site. Testing data included 40 simulated anomalies (20 of each type) and 20 unseen normal records.

**Table 2:** Model results

			Parameters									
Site	Data type	Technique	a	b	ν	μ	τ	θ	F1 (mean ± SD)	S_normal_	S_anomalies_	S
Brain	Training	3D	0.04	0.63	0.02	0.02	0.19	0.24	0.94 ± 0.01	40	60	1003
		SRT	0.10	0.47	0.01	0.01	0.38	0.05	0.92 ± 0.02	40	60	1103
		IMRT	0.02	1.12	0.01	0.01	0.13	0.45	0.98 ± 0.01	40	60	1868
	Testing	3D	0.04	0.63	0.02	0.02	0.18	0.25	0.94	20	40	1043
		SRT	0.10	0.47	0.01	0.01	0.38	0.05	0.90	20	40	1143
		IMRT	0.02	1.12	0.01	0.01	0.13	0.45	0.99	20	40	1908
Thoracic	Training	3D	0.05	0.53	0.02	0.01	0.22	0.44	0.93 ± 0.2	40	60	404
		SBRT	0.07	0.49	0.05	0.01	0.22	0.47	0.97 ± 0.01	40	60	611
		IMRT	0.02	0.19	0.018	0.03	0.12	0.30	0.93 ± 0.02	40	60	712
	Testing	3D	0.05	0.53	0.018	0.01	0.21	0.43	0.95	20	40	444
		SBRT	0.07	0.49	0.05	0.01	0.22	0.47	0.95	20	40	651
		IMRT	0.02	0.19	0.02	0.03	0.12	0.29	0.90	20	40	752

3D = 3-dimensional; IMRT = intensity modulated radiation therapy; S = number of historical records for the technique for each treatment site; S_anomalies_ = number of simulated anomalies in each training or testing set; SBRT = stereotactic body radiation therapy; S_normal_ = number of normal records used in the training or test set; SRT = stereotactic radiotherapy.

F1 scores for training and testing are shown in the 10th column for each technique within each model. Standard deviations in training sets are found over 50 runs. Columns 4 to 9 show model parameters, which are as follows: a and b are optimization parameters for setting flagging thresholds (t_F_ and t_Rx_, respectively). ν and μ are percentages of the historical database for which the R and F statistics were generated, and τ and θ are mean pairwise feature and prescription distances.

### Model performance across different treatment sites

**Figure 1:**
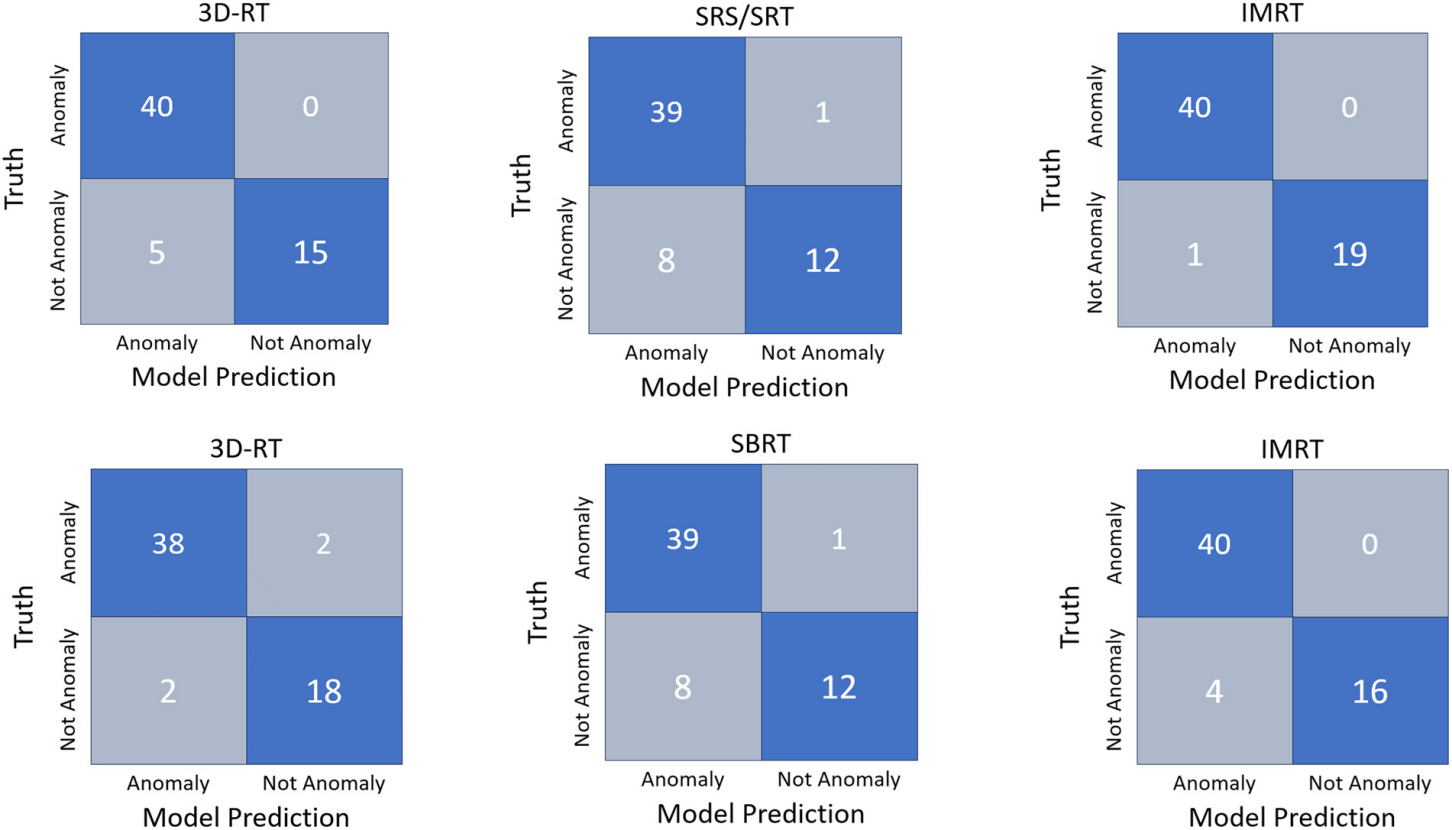
Model classifications. Confusion matrices for the brain model (top) and thoracic model (bottom) are shown. Each 2 × 2 matrix represents the classification performance of the model, where the x-axis corresponds to the model predicted labels (anomaly or not anomaly) and the y-axis represents the truth labels. The 4 quadrants indicate the number of correct prediction (true positives and true negatives in blue) and incorrect predictions (false positives and false negatives in gray). *Abbreviations:* 3DRT = 3-dimensional conformal radiation therapy; IMRT = intensity modulated radiation therapy; SBRT = stereotactic body radiation therapy; SRT = stereotactic radiotherapy; SRS = stereotactic radiosurgery.

The thoracic model achieved high F1 scores when testing both training data and test data. The scores were as follows: for IMRT, 0.93 for training data and 0.90 for test data; for SBRT, 0.97 for training data and 0.95 for test data; and for 3DRT, 0.93 for training data and 0.95 for test data. Classification results for the brain model are illustrated in Fig. 1. Notably, the model had a high success rate in predicting anomalies, with few misclassifications.

Similarly, in the brain model, the F1 scores were strong. The scores were as follows: for IMRT, 0.98 for training data and 0.99 for test data; for SRT, 0.92 for training data, and 0.90 for test data; and for 3DRT, 0.94 for training data and 0.94 for test data. Binary classification of the thoracic model’s test data is illustrated in Fig. 1, revealing good performance for IMRT and 3DRT, with a few normal records incorrectly flagged as anomalies. However, SBRT showed more misclassifications with 8 normal records predicted as anomalies.

### Physician validation of model predictions

Our collaborating RO reviewed a set of 30 model-flagged anomalies (correctly flagged anomalies and falsely flagged normal records) per site model. For the thoracic model, 18 out of 20 correctly flagged anomalies were confirmed to be anomalies. Two of the 10 incorrectly flagged normal records were identified by the oncologist to be valid flags and/or appropriate for review. For the brain model, 18 of 20 correctly flagged anomalies were confirmed to be anomalies, and 3 of 10 incorrectly flagged normal records were denoted as accurate flags and/or appropriate for review.

### Statistical analysis

For our statistical analysis of the comparison of the model’s predictions to the ground truth, we found the χ^2^ statistics of 3.81 to be less than the critical value of 3.84 when using an alpha value of 0.05, meaning that we will keep our null hypothesis, and assume that the model’s predictions and ground truth are not significantly different.

### Secular time analysis

Our linear scaling approach for incremental learning was used as a proof of concept in this study, and the results show that there is a secular time trend in our data, as F1 for the unscaled data set was 0.9 and increased to 0.95 when the scaled data set was used to give a higher weight to more recent treatment records.

**Figure 2:**
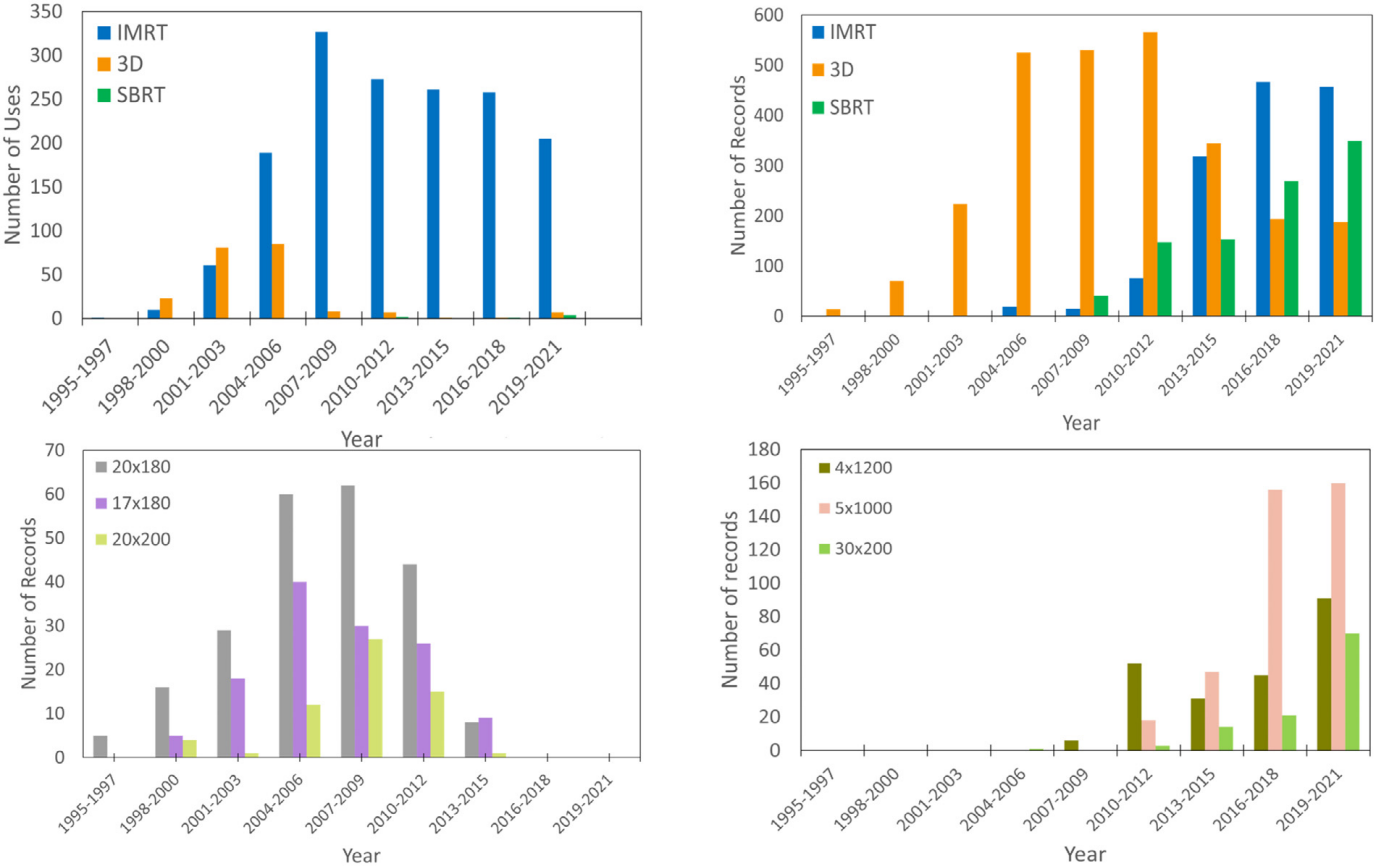
Evolutionary trends. Advances in technology and treatment protocols have led to a shift from 3-dimensional conformal radiation therapy (3DRT) to intensity modulated radiation therapy (IMRT) and stereotactic body radiation therapy (SBRT). The top-left plot illustrates the transition from 3DRT to IMRT for prostate radiation therapy. The top-right plot highlights the predominance of IMRT in thoracic treatments since the early to mid-2010s, alongside a continuous upward trend in SBRT use, while 3DRT has steadily declined. The bottom-right plot depicts the increasing adoption of 3 radiation therapy prescriptions for thoracic treatments: 2 SBRT regimens (4 fractions of 1200 cGy and 5 fractions of 1000 cGy) and an IMRT regimen (30 fractions of 200 cGy), all trending upward from the early 2010s to 2021. In contrast, the bottom-left plot shows the decline of outdated 3DRT prescriptions (20 fractions of 180 cGy, 17 fractions of 180 cGy, and 20 fractions of 200 cGy) from the early to mid-2010s, reflecting a shift toward updated dosage schemes at the institution.

New protocols and advancements in the field cause changes in the method of radiation delivery and dosage schemes over time,^27^, ^28^, ^29^ which contribute to the secular trend seen in our linear scaling approach. This led us to further analyze the trends in our data, and the clear evolutionary trends for RT techniques and dosage schemes are shown in Fig. 2. For thoracic treatments, we see an almost sole use of 3-dimensional (3D) treatments until the late 2000s, with a steep decline in subsequent years, and IMRT and SBRT quickly becoming the favored techniques. Similarly, prostate treatment sites follow the same trend. Three-dimensional was the preferred treatment technique until an almost exponential increase in the use of IMRT in the mid-2000s. This trend is influenced and coincides with studies such as Michalski et al’s^30^ analysis of the preliminary toxicities of 3D and IMRT, and others.^31^, ^32^, ^33^, ^34^ The trend of increasing use of IMRT and SBRT techniques is evident in Rx usage. Our collaborating physician identified the current commonly used Rx and retired Rx for the thoracic data sets. Three Rx rising in use are also depicted in Fig. 2; all 3 were not commonly used until the mid-2010s. On the other hand, Rx, whose use has ceased in recent years, are also shown. Moreover, 3D Rx in the mid to late 2000s were common; however, these Rx ceased to be used for treatments in the mid-2010s.

## Discussion

In this study, we validated a data-driven distance model that uses historical data to determine distances between Rx and feature sets for records with similar Rx. This study, along with our original work,^21^ is among the first on automatic Rx error detection in RT. Related works by Sharabi et al^35^ and Pashtan et al^36^ investigate automated RT Rx error detection; however, their studies employ different detection methods. Sharabi et al.’s^35^ method of Rx error detection cross-references new Rx with the frequency of previously treated Rx. If the new Rx was not previously seen or infrequently used, then it was flagged. We believe that conditional probability is a valid approach; however, it is very labor-intensive as it would require calculating the conditional probability for every feature combination. There has been no additional published information since 2012. Pashtan et al’s^36^ method incorporates rule-based error detection, where rules defining appropriate RT Rx were established. When a new Rx did not fall within the bounds of the rules, an automatic email was generated and sent to the prescriber. This method only looks at the Rx’s fractional and total dose and does not analyze patient-specific features. Expanding on this method could involve the generation of rules for every feature; however, this would be very time-consuming and would need to be adapted as RT regimens change. Our approach, a data-driven model, is much simpler than the alternative methods. The detection model is able to calculate appropriate thresholds for flagging, and with the addition of incremental learning, the model can adapt to new RT regimens. Additionally, our model is intuitive, as its predictions show why the Rx was flagged. Examples of the model predictions are seen in Table 3, where the model output gives the prediction, truth, and type of anomaly (if applicable).

**Table 3:** Model prediction examples

												Metrics				
Record #	Fx	Dose per Fx (cGy)	Technique	Energy (MV)	Rx IDL (%)	Intent	Diagnostic code	Age group	Truth	Prediction	Type	R	t_Rx_	F	t_F_	# Records in DB
1	1	150*	SRT	1.25	50	Curative	C79.31	Adult	1	1	1	0.596	0.005	-	-	0
2	3*	2000	SRT	6x	50	Palliative	C79.31	Adult	1	1	1	0.5	0.005	-	-	0
3	3	600*	SRT	6x	82	Palliative	C79.31	Adult	1	1	1	0.059	0.005	-	-	0
4	7*	300*	SRT	6x	82	Palliative	C79.31	Adult	1	1	1	0.514	0.05	-	-	0
5	1	1500	SRT	6x	100*	Palliative	C79.31	Adult	1	1	2	0	0.005	0.364	0.180	45
6	1	1800	SRT	1.25	50	Palliative	C79.31	Pedi*	1	1	2	0	0.005	0.202	0.180	177
7	10	100*	3D	6x	99	Palliative	C79.31	Adult	1	1	1	0.386	0.010	-	-	0
8	12	520*	3D	6x	98	Palliative	C79.31	Adult	1	1	1	0.625	0.010	-	-	0
9	4*	180	3D	6x	100	Curative	C71.3	Pedi	1	1	1	0.043	0.010	-	-	0
10	10	300	3D	4e*	90*	Palliative	C69.31	Adult	1	1	2	0	0.010	0.4	0.112	519
11	8	300	3D	10x	95	Palliative	C71.9*	Adult	1	1	2	0	0.010	0.507	0.112	16
12	5	180	3D	6x	100	Palliative*	C71.7	Pedi	1	1	2	0	0.010	0.297	0.112	23
13	20*	300	IMRT	6x	100	Palliative	C79.31	Adult	1	1	1	0.322	0.009	-	-	0
14	25*	150*	IMRT	10x	100	Palliative	C79.31	Adult	1	1	1	0.25	0.009	-	-	0
15	32*	200	IMRT	6x	100	Curative	C71.1	Adult	1	1	1	0.067	0.009	-	-	0
16	10	300	IMRT	6x	100	Curative	C71.9	Pedi*	1	1	2	0	0.009	0.525	0.148	52
17	5	180	IMRT	6x	100	Palliative*	C71.1	Adult	1	1	2	0	0.009	0.202	0.148	142
18	25	180	IMRT	6x	70*	Palliative*	C71.2	Adult	1	1	2	0	03009	0.4	0.148	34
19	4	2000*	SBRT	6x	84	Curative	C34.11	Adult	1	1	1	0.559	0.031	-	-	0
20	5	1800*	SBRT	6fff	84	Curative	C34.11	Adult	1	1	1	0.75	0.031	-	-	0
21	3	1800	SBRT	6fff	88	Curative	C34.32	Pedi*	1	1	2	0	0.031	0.304	0.104	91
22	3	1800	SBRT	6fff	83	Curative	C15.3*	Adult	1	1	2	0	0.031	0.132	0.104	91
23	20*	100*	3D	10x	97	Curative	C34.31	Adult	1	1	1	0.901	0.021			0
24	10	220*	3D	10x	100	Palliative	C34.10	Adult	1	1	1	0.100	0.021			0
25	30*	100*	3D	10x	91	Palliative	C34.31	Adult	1	1	1	1.677	0.021			0
26	10	200	3D	10x	100	Curative	C34.80	Pedi*	1	1	2	0	0.021	0.253	0.112	20
27	10	300	3D	10x	98	Palliative	C22.8*	Adult	1	1	2	0	0.021	0.012	0.112	252
28	15	250	3D	10x	98	Curative*	C34.10	Adult	1	1	2	0	0.021	0.221	0.112	75
29	52*	180	IMRT	10x	100	Curative	C15.4	Adult	1	1	1	0.5	0.007	-	-	0
30	18*	250*	IMRT	6x	100	Curative	C15.4	Adult	1	1	1	0.486	0.007	-	-	0
31	20*	250*	IMRT	6x	100	Curative	C34.12	Adult	1	1	1	0.453	0.007	-	-	0
32	23	180	IMRT	6x	100	Palliative*	C15.4	Adult	1	1	2	0	0.007	0.243	0.022	20
33	32	180	IMRT	6x	100	Curative	C15.5*	Adult	1	1	2	0	0.007	0.33	0.022	25
34	25	180	IMRT	6x	100	Curative	C15.5	Adult	0	0	-	0	0.007	0	0.22	136
35	23	200	IMRT	6x	100	Curative	C71.1	Adult	0	0	-	0	0.009	0	0.148	535
36	10	300	3D	6x	97	Palliative	C79.31	Adult	0	0	-	0	0.010	0	0.112	519
37	5	1000	SBRT	6fff	86	Curative	C15.3	Adult	1*	0*	-	0	0.031	0.061	0.104	377
38	25	200	3D	6x	97	Curative	C71.1	Adult	0*	1*	1	0.145	0.010	-	-	10
39	25	180	IMRT	10x	100	Curative	C15.4	Adult	0*	1*	2	0	0.007	0.067	0.022	134
40	30	180	IMRT	6fff	100	Curative	C71.8	Adult	0*	1*	2	0	0.009	0.205	0.148	68

3D = 3-dimensional; F = average feature distance; IMRT = intensity modulated radiation therapy; Pedi = pediatric; R = average prescription distance; Rx = prescription; SBRT = stereotactic body radiation therapy; t_F_ = feature threshold for flagging; t_Rx_ = prescription threshold for flagging; IDL = Isodose line.

⁎ The error that occurs in the data. Displayed are examples of model predictions. The table shows the Rx (dose per fraction and number of fractions) along with the features used in this study (technique, energy, Rx isodose line, intent, diagnostic code, and age group). The truth column tells if the record was a normal record (0) or a simulated anomaly (1). The prediction column is the model’s prediction for that record, and the type of anomaly that it predicted (1 being an Rx anomaly or 2 being a feature anomaly). The last column shows the number of times that the Rx is seen in the historical database. The first record shows a Rx anomaly, where the model prediction (1) matches the truth (1) for a Rx type anomaly (1), because the R statistic exceeds the Rx threshold. It is seen that the Rx is not used in the historical database. Record 5 is an example of a correctly predicted feature type anomaly, where the t_Rx_ is not exceeded by the R statistic; however, t_F_ is exceeded by the F statistic. The given records show correctly predicted atypical records (records 1-33), normal records not flagged by the model (records 34-36), and incorrectly predicted records (records 37-40).

We aimed to assess the model's performance across different treatment sites and institutions. F1 scores for training data and test data for both treatment sites exceeded 0.90, as shown in Table 2, showing high stability for the model when applied to multiple sites. Additionally, this study’s results for the thoracic model show strong robustness across multiple institutions' data, as our results show similar model performance metrics, with this study scoring slightly higher for all 3 techniques when compared to our original work.^21^ This improvement was partly because of enhanced data engineering methods, such as adjusting how the model handled diagnostic codes and age categories, along with data imputation to fill missing values in nearly complete features. This resulted in data sets without missing values.

### Limitations

In our methods, we aimed to remove anomalous records; however, some anomalies may remain, potentially affecting the model's accuracy. Averaging the parameters n and m, as discussed in our original work, can help mitigate this error.

Our data limitations arise from a restricted timeframe (1995 to 2021), in that we are missing current data reflecting recent protocol changes, which is evident in the prostate treatment site.^37^ A physician specializing in prostate RT treatments reviewed Rx data from our query and concluded that the data was out of date, as many current prostate treatments follow simultaneous integrated boost protocols.^38^^,^^39^ Additionally, we recognize several factors that may have influenced our results. The 2 institutions included in this study represent significantly different clinical environments, which may introduce systematic biases in Rx patterns. The first institution, a large academic center, features physicians from diverse training backgrounds with varying levels of experience, leading to greater variability in Rx practices. This center also serves as a demographically diverse patient population from multiple states, which may impact treatment decisions. In contrast, the second institution is a private clinic with physicians from more uniform training backgrounds and a more localized, homogeneous patient population, potentially leading to more standardized Rx behaviors. Furthermore, differences in treatment capabilities, such as the absence of a linac-based stereotactic radiosurgery (SRS) program at the first institution and the specialization in this technology at the second, may further contribute to variability in treatment approaches. These factors should be considered when interpreting our results.

The model is generalizable based on our validation results, but requires physician and data modeling expert input. Although this current study focuses on testing the model within a controlled environment with simulated anomalies and retrospective data only, we recognize that using real anomalies would be ideal for better capturing the complexity of real-world scenarios and is a necessary next step. Transiting from a controlled testing environment to live application will pose additional challenges that are beyond our study’s scope, such as adapting the model to diverse, dynamic clinical environment, patient-specific variations, extensive data collection on real-life anomalies and collaborations between various team members. We plan to address these challenges in a future study specifically through live clinical trials, which will assess the model's performance with actual patient data in real-time settings. These trials are essential to further validate the model’s adaptability and generalizability in clinical environments.

**Figure 3:**
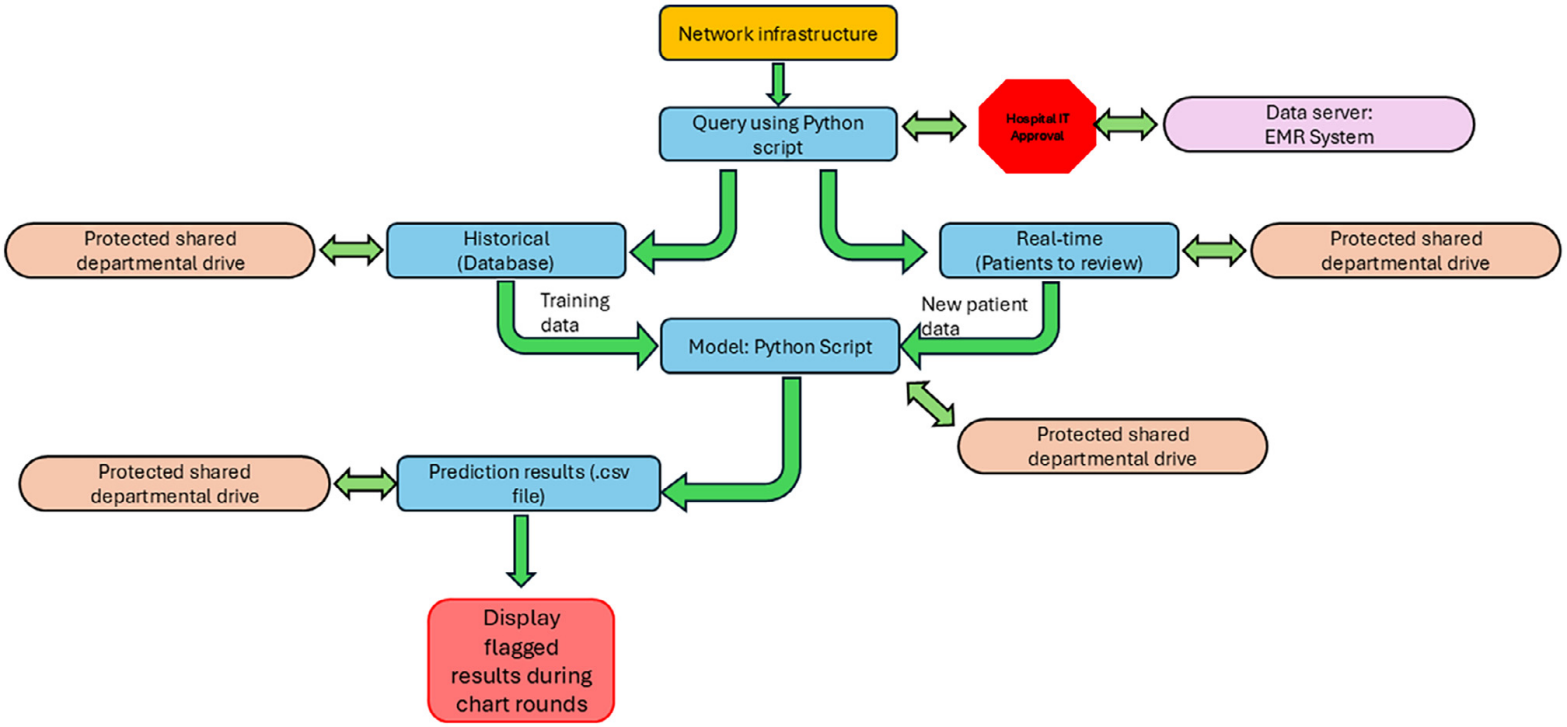
Integration into hospital infrastructure. Use of the model starts with the hospital network infrastructure. The infrastructure should include all required components to functionally use an electronic medical record (EMR) system (Wi-Fi, routers, personal computers connected to the network, and secure data transfer pathways [displayed as arrows]). In addition, the infrastructure should include shared departmental drives/folders that are secured with password protection to maintain patient confidentiality and data integrity, which are visualized as orange ovals. To query data, IT administration will need to approval and assist with the backend of the EMR system in order to run the python script to extract the necessary data from the secure data server, depicted in lilac. Our query has 2 components. The first is a historical database query, where we obtain data for training purposes. The second type of query would be used for real-time, new patient, data acquisition. The data obtained during both types of queries must be saved on protected shared drives. Next, a python script will be executed that will train and store the model in a protected departmental drive. The python script will then use the new patient data and the trained model to predict anomalies. The output of the model, in the form of a .csv file, will be saved to the protected departmental drive. For clinical use, a network PC can display the .csv file during chart rounds.

Implementing a new technological tool in clinical practice can be challenging and worrisome. Figure 3 provides a detailed workflow diagram that illustrates our approach in implementing the system based on a real-life scenario and the technological infrastructure required for clinical application of our model while complying with health care regulations.

Our method of validation does not resemble peer review, as peer review is a collaborative process that relies on discussion among experienced peers to determine the correctness of a patient’s Rx. However, a benefit of this method is that we were able to compare the model’s predictions to that of a single physician for atypical records, which allowed the physician to confirm that the simulated anomalies closely mimicked the real anomalies and were realistic. This also enabled us to conduct an error analysis, identifying the shortcomings of our model. Among the model predictions reviewed by the collaborative RO, all incorrect flags were related to features. The RO deemed less than 3 out of 10 predictions per model as atypical, indicating that the model tends to over-flag potential feature anomalies. This suggests there is room for future improvement and refinement of feature data.

### Incremental learning

Acknowledgment of the outdated and rising Rx is vital to the success of the model. The inclusion of outdated Rx in the training data allows for the possibility of anomalies to go unflagged, meaning outdated Rx could be seen as typical. On the other hand, records with new Rx may be flagged as anomalies, because of the lack in the number of records. We propose several incremental learning strategies. First, we limit the use of data that are too old, such as data from more than 2 decades ago. Another option is to use a sliding window approach, where data are fed into the model every 2 years, and the oldest 2 years are retired. These approaches have not been tested in the current study but offer opportunities for future work.

To ensure model relevance, we emphasize the use of automated data collection and preprocessing systems, along with automation for simulating anomalies. We propose a structured approach for maintaining the model's clinical relevance by regularly integrating updates from major RT protocol changes, clinical trial results, and radiation safety guidelines. Professional organizations like the American Society for Radiation Oncology and the European Society of Radiotherapy and Oncology update clinical guidelines every 3 to 5 years, while regulatory bodies periodically revise safety protocols. To ensure the model stays current, we recommend formal reviews and retraining at least twice a year, with interim updates if significant changes occur. Continuous input from clinicians will also guide these updates, ensuring that practical insights from clinical practice are incorporated.

## Conclusions

In conclusion, we have shown that the automated Rx anomaly tool generalizes well to a second institutional database and multiple treatment sites which highlights the potential usefulness of the tool as more generally adoptable for detecting Rx anomalies in RT, given the stated limitations.
